# Invasive *Impatiens glandulifera*: A driver of changes in native vegetation?

**DOI:** 10.1002/ece3.7135

**Published:** 2021-01-12

**Authors:** Judith Bieberich, Stefanie Müller, Heike Feldhaar, Marianne Lauerer

**Affiliations:** ^1^ Ecological Botanical Gardens Bayreuth Center for Ecology and Environmental Research (BayCEER) University of Bayreuth Bayreuth Germany; ^2^ Animal Ecology 1 Bayreuth Center for Ecology and Environmental Research (BayCEER) University of Bayreuth Bayreuth Germany

**Keywords:** causality of impact, context‐dependency, plant invasion, planting experiment, removal experiment, riverside vegetation

## Abstract

Biological invasions are one of the major threats to biodiversity worldwide and contribute to changing community patterns and ecosystem processes. However, it is often not obvious whether an invader is the “driver” causing ecosystem changes or a “passenger” which is facilitated by previous ecosystem changes. Causality of the impact can be demonstrated by experimental removal of the invader or introduction into a native community. Using such an experimental approach, we tested whether the impact of the invasive plant *Impatiens glandulifera* on native vegetation is causal, and whether the impact is habitat‐dependent. We conducted a field study comparing invaded and uninvaded plots with plots from which *I. glandulifera* was removed and plots where *I. glandulifera* was planted within two riparian habitats, alder forests and meadows. A negative impact of planting *I. glandulifera* and a concurrent positive effect of removal on the native vegetation indicated a causal effect of *I. glandulifera* on total native biomass and growth of *Urtica dioica*. Species α‐diversity and composition were not affected by *I. glandulifera* manipulations. Thus, *I. glandulifera* had a causal but low effect on the native vegetation. The impact depended slightly on habitat as only the effect of *I. glandulifera* planting on total biomass was slightly stronger in alder forests than meadows. We suggest that *I. glandulifera* is a “back‐seat driver” of changes, which is facilitated by previous ecosystem changes but is also a driver of further changes. Small restrictions of growth of the planted *I. glandulifera* and general association of *I. glandulifera* with disturbances indicate characteristics of a back‐seat driver. For management of *I. glandulifera* populations, this requires habitat restoration along with removal of the invader.

## Graphical abstract

In invasion biology, causality of impact is often not obvious. By experimental removal and planting the plant invader *Impatiens glandulifera,* we showed that it had a low but causal negative impact on native European vegetation in riparian meadows and alder forests. Thus, *I. glandulifara* has some characteristics of a driver of ecosystem changes.

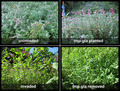

## 1 INTRODUCTION

Biological invasions are an important aspect of anthropogenic global change and are considered to be one of the major threats to biodiversity worldwide (Sala et al., 2000). A well‐documented impact of species invasions is to reduce native biodiversity, species abundances, change community patterns, and ecosystem processes such as nutrient cycling in invaded communities (Dogra et al., 2010; Ehrenfeld, 2010; Vilà et al., 2011)⁠. However, it is difficult to disentangle cause and effect of an invasion. An alien species can invade an intact ecosystem and cause changes there, thus be the “driver” of the changes (Bauer, 2012; Didham et al., 2005; MacDougall & Turkington, 2005)⁠. Alternatively, invasion may be facilitated by earlier ecosystem changes, such as global warming, land use change, or disturbances. Then the invasion is only a symptom, and the invader a “passenger” of the underlying change (Bauer, 2012; Didham et al., 2005; MacDougall & Turkington, 2005)⁠. Drivers and passengers are the extreme positions of a continuum, and several invasive species rather fall in‐between those categories (Bauer, 2012)⁠. Such “back‐seat drivers” benefit from previous changes, but once established they become drivers of further changes (Bauer, 2012). Another challenge in assessing the impact of an invader are context‐dependencies. Invasion can, for example, depend on ecosystem, invasion stage, or species traits (Kueffer et al., 2013)⁠. The more an invader is a passenger of changes, characteristics of the native ecosystem such as habitat conditions and species composition of the receiving community should influence the outcome of invasion and lead to differences between habitats. Invasion of a passenger is rather unlikely the more it relies on previous ecosystem changes. Knowledge about driver and passenger characteristics of an invader and context‐dependencies is important to understand invasion processes and to develop more targeted management plans.

*Impatiens glandulifera* originating from the Himalaya mountains is a very common invasive species in Central Europe. Rapid spread and population growth of this annual species are enabled by a large number of seeds and their effective dispersal. Seeds are catapulted over several meters due to an explosion mechanism of the capsule and subsequently often transported by water flows (Beerling & Perrins, 1993). *I. glandulifera* invaded various wet habitats such as mesotrophic grass‐ and woodlands but increasingly also forests and ruderal sites outside of the riparian zone (Beerling & Perrins, 1993; Čuda et al., 2017; Čuda et al., 2020; Pyšek & Prach, 1993, 1995). *I. glandulifera* is capable of suppressing native plants because of a high competitive effect along with a vigorous growth and the release of allelopathic substances such as 2‐methoxy‐1,4‐naphthoquinone as shown in experimental studies (Bieberich et al., 2018; Gruntman et al., 2014; Loydi et al., 2015; Power & Sánchez Vilas, 2020; Ruckli et al., 2014; Vrchotová et al., 2011). Another factor benefiting *I. glandulifera* is, for example, release from natural enemies such as insect herbivores and parasitic rust fungi (Tanner et al., 2014). Under field conditions, it can form dominant stands with a height of up to three meters (Beerling & Perrins, 1993; Bieberich et al., 2020). Nonetheless, the impact of *I. glandulifera* on native plant communities is rated ambiguously, and it is not clear whether the impact is causal, thus *I. glandulifera* being a driver of ecosystem changes. Comparing invaded and uninvaded sites Hejda and Pyšek (2006), Hejda et al., (2009), and Diekmann et al., (2016) found only weak, but Kiełtyk and Delimat (2019) found strong differences of plant diversity and composition. From a previous study, we know that *I. glandulifera* and native vegetation cover correlated negatively, and the correlation depended on environmental conditions at a particular site (Bieberich et al., 2020)⁠. However, with these observational approaches, causality of impact is difficult to address (Hejda & Pyšek, 2006; Kumschick et al., 2015; Stricker et al., 2015). Some studies—also with ambiguous results—experimentally removed the invader *I. glandulifera* (Cockel et al., 2014; Čuda et al., 2017; Hejda & Pyšek, 2006; Hulme & Bremner, 2006)⁠. Such removal experiments can help to identify whether an effect is causal (Kumschick et al., 2015; MacDougall & Turkington, 2005)⁠. If the invader is a driver of changes, removal should rescue the state prior to invasion. However, also removal experiments have some drawbacks (Hulme & Bremner, 2006; Kumschick et al., 2015; Stricker et al., 2015)⁠. Response of the native community could also be caused by the disturbance of the treatment itself. Removal of any other, even native, species could have the same effect, for example, because this may lead to higher resource availability. The process of native community recovery could also take longer time than the study, and thus effects may not become visible yet, especially if there are legacy effects of the invasion. An effective method to study causal effects is to add the invader to the native community (Stricker et al., 2015)⁠. However, this is rarely implemented under field conditions because then, a careful handling of the invader is required.

The aim of this study was to investigate whether *I. glandulifera* has a causal negative impact on the native vegetation and whether this impact depends on the habitat. Due to its uneven distribution within one field site, *I. glandulifera* can be transplanted from an invaded patch into an uninvaded patch, without introducing the species to a new site. To disentangle cause and effect of invasion, we combined the classical approaches to compare invaded and uninvaded patches, and to remove *I. glandulifera* from invaded patches, with transplanting *I. glandulifera* into uninvaded patches. Thus, the transplanting represents a control for removal and vice versa. To test for habitat‐dependence, we replicated this experimental approach in two different riverside habitat types, alder forests and meadows. We expect that *I. glandulifera* has a negative impact on the native vegetation, specifically on α‐diversity, biomass and species composition of the resident vegetation, and on individual plant growth of resident species. For the latter, *Urtica dioica* was chosen as target species because it is one of the most frequent native co‐occurring species of *I. glandulifera* in both habitats. Because of the high competitive and allelopathic effect of *I. glandulifera* on neighboring plants, especially native plant growth should be affected even within a short time leading to changed species abundances and plant performance at the spatial scale of the experimental plots. If *I. glandulifera* is a driver of changes having a causal impact, (a) removal of *I. glandulifera* is expected to have a positive (recovery) effect on the native vegetation, and (b) planting *I. glandulifera* into formerly uninvaded plots should have a negative impact on the native vegetation. Additionally, (c) establishment of planted *I. glandulifera* and impact of planting and removal are expected to depend on the habitat because plant growth and species interactions are shaped by environmental conditions. If *I. glandulifera* has no causal impact on the resident vegetation, its removal should have no recovery effect, and its planting should have no negative impact on the resident vegetation. The native vegetation could still differ between invaded and uninvaded patches if *I. glandulifera* has no causal impact but is only a passenger of changes.

## 2 MATERIALS AND METHODS

### 2.1 Implementation of the field experiment

Field studies were conducted at four riverside sites around Bayreuth, Germany, also used in a previous study (Bieberich et al., 2020)⁠. Among them were two open sites comprised of abandoned meadows with tall herbaceous vegetation (Waischenfeld 49°49.98′N 11°20.17′E, Weidenberg 49°56.95′N 11°42.15′E) and two alder swamp forests, also with tall herbaceous vegetation (Ludwigschorgast 50°6.66′N 11°35.20′E, Neunkirchen 49°55.20′N 11°38.05′E). Each site consisted of a mosaic of patches with and without *I. glandulifera*.

**FIGURE 1:**
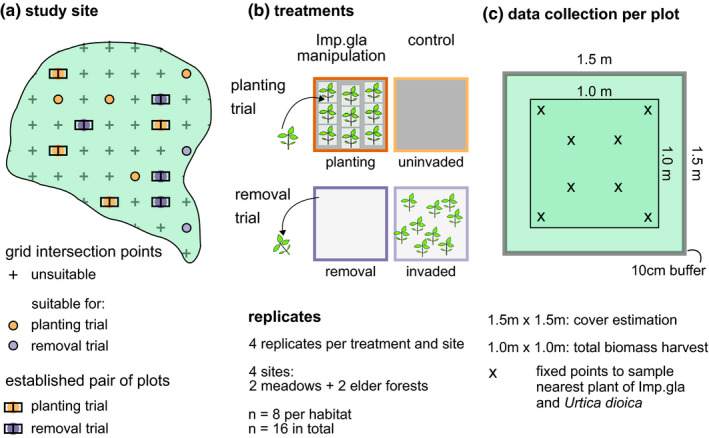
Study design. (a) Concept of selection of suitable plot positions along a grid laid over each study site. From the suitable positions, it was randomly chosen which positions were used to either conduct the planting or the removal trial there. (b) In total, there were four treatments: planting of *I. glandulifera* within uninvaded plots and a paired uninvaded control, removal of *I. glandulifera* from invaded plots and a paired invaded control. (c) Data collection within each plot: cover and vegetation height were gathered within the total 2.25 m^2^ plot; total biomass was harvested within the central 1 m^2^; individual plants of *I. glandulifera* and *U. dioica* were harvested within the total plot except a 10‐cm buffer margin, whereby those eight plants being nearest to the eight fixed points in the plot were sampled

To choose positions for the plots, a grid of 20 m × 20 m was laid over each study site (Figure 1a), ten meters shifted to the grid of our previous study (Bieberich et al., 2020)⁠. In March to April 2016, all grid intersection points were checked for suitability to conduct either removal or planting of *I. glandulifera* there (Figure 1a). Suitability was predefined as an area of 2 m × 4 m homogeneous herbaceous vegetation, in spring either invaded by *I. glandulifera* with 5%–40% cover for the removal trial or uninvaded with a maximum of five *I. glandulifera* plants for the planting trial. Additionally, suitable positions in alder forests had to have a more or less closed canopy and positions in meadows had to be not covered by trees as far as possible. Out of all suitable positions, four positions per study site and trial (planting, removal) were randomly chosen for usage. On each chosen position, a pair of 1.5 m × 1.5 m plots was established with a gap of 0.5 m between the single plots. One randomly chosen plot of the pair was left unchanged either as an invaded control or an uninvaded control, respectively (Figure 1b). Within the second plot of the pair, occurrence of *I. glandulifera* was manipulated in May (2016–05‐09/27). For the removal treatment, all *I. glandulifera* plants were removed. Plots were checked and, if necessary, removal repeated every other week for the first 2 months and then at larger intervals since only few *I. glandulifera* plants emerged. Initially removed *I. glandulifera* had a stem length of 21 ± 12 cm mean ± *SD* (*n* = 65 with five plants randomly chosen and measured per plot) and in total 6–87 g dry biomass of *I. glandulifera* was removed per plot (mean 26 g, *n* = 13 plots). For the planting treatment, 63 *I. glandulifera* plants, corresponding to about 5%–10% cover in spring, were transplanted into each plot with always 20 cm distance between individual plants (mean stem length 19 ± 5 cm, *n* = 65 with five plants randomly chosen and measured per plot). Transplanted individuals were always collected and transplanted within the same study site. After about 10 days, we checked whether the transplanted individuals had grown and replaced failed individuals once. We wanted to achieve that the uninvaded plots and plots where *I. glandulifera* was removed were free of *I. glandulifera* over summer, while naturally growing and planted *I. glandulifera* developed 15%–75% cover. This moderate cover of *I. glandulifera* was aimed for because a very high cover of *I. glandulifera* in the removal trial could make the measurement of a recovery effect difficult. For a recovery effect in particular, a certain amount of native vegetation is required. During summer, in total three pairs of plots belonging to the removal trial were destroyed by fallen trees and wild boars in three different study sites. This resulted in *n* = 13 for the removal trial and *n* = 16 pairs of plots for the planting trial.

Effect of the *I. glandulifera* manipulations on the resident vegetation was evaluated in autumn 2016. To study the effect on traits of individual plant growth, *U. dioica* was chosen as native target species because it was the only species that was sufficiently abundant in all sites and treatments. When *I. glandulifera* or *U. dioica* occurred in a plot, their cover was estimated within the total plot on 2016–08‐19/25 according to extended Braun‐Blanquet scale which was afterward converted into the numerical values 0, 0.1, 0.5, 2.5, 8.8, 20.5, 37.5, 62.5, 87.5 percent cover (Braun‐Blanquet, 1964; Reichelt & Wilmanns, 1973)⁠. Data on vegetation height and biomass were collected on 2016–08‐30/10‐04. Maximum vegetation height of the resident vegetation was recorded with a folding ruler as mean height of the five highest plants in the plot, regardless of plant species. In one pair of plots, the plants were not totally upright due to rainfall or wind, and thus we could not measure vegetation height. When occurring, eight individual plants each of *I. glandulifera* and *U. dioica* were harvested per plot. The respective plants nearest to one of eight fixed points in the plot were chosen whereby the outermost 10 cm margin of the plot was ignored (Figure 1c). In some cases, there were only six or seven plants of *U. dioica* in a plot, and accordingly sample size decreased. Of each sampled plant, stem length was measured with a folding ruler and dry weight of the vegetative plant parts and the infructescence were measured separately. Within the central 1 m^2^ of each plot, all vascular plants were harvested and the biomass sorted by species. Species were determined using standard literature (Eggenberg & Möhl, 2013; Jäger, 2017; Jäger et al., 2013; Schmeil et al., 2011),⁠ and total dry weight was recorded per species. To measure dry weight, all plant material was dried at 90°C for 2 days and weighed to the nearest 0.01 g (weighing scale Mettler PM 4,600). Thus, all biomass data, hereafter, are given as dry mass.

### 2.2 Statistical analysis

All statistical analyses were done with the software package R 4.2.0 (2020–06–22), R Studio 1.3.959. In addition to the core packages, lme4 (Bates et al., 2015), vegan (Oksanen et al., 2019), car (Fox & Weisberg, 2019), and broom (Robinson et al., 2020)⁠ were used for statistical analyses, plyr (Wickham, 2011), dplyr (Wickham et al., 2020),⁠ and reshape2 (Wickham, 2007)⁠ for data handling, ggplot2 (Wickham, 2016), cowplot (Wilke, 2019),⁠ and RColorBrewer (Neuwirth, 2014)⁠ for visualization. Linear models were used to test whether total biomass, cover, individual plant biomass, and stem length of *I. glandulifera* depended on whether *I. glandulifera* was planted or grew naturally and whether in the habitat meadow or alder forest. In the case of individual plant biomass and stem length (*n* = 8 per plot) plot was applied as random factor. Species number, Shannon index, total biomass, and vegetation height of the resident vegetation (all species except *I. glandulifera*) were compared between invaded and uninvaded control treatments and between habitats using linear models. Likewise, total biomass, cover, stem length, vegetative biomass, and infructescence biomass of *U. dioica* were compared between invaded and uninvaded control situations with linear models, and additionally total biomass of the most frequent native species with Mann‐Whitney‐*U* tests. However, habitat‐dependency could not be tested with these parameters because sample size per habitat was too low. In the case of individual plant growth of *U. dioica* mean values per plot were used making the use of plot as random factor unnecessary. For all linear models, either pair of plots or study site was applied as random factor whenever possible. In some cases, it was not possible to use the random factor because its variance was estimated zero. Use of error distribution family was decided per parameter based on visual inspection of the model residuals, resulting in generalized linear models where necessary.

To quantify impact intensity of manipulation of *I. glandulifera* within each pair of plots, the relative interaction index RII was calculated (Armas et al., 2004; Gruntman et al., 2014) comparing manipulation and control, according to the equation (manipulation ‐ control)/(manipulation + control). RII is bound to the range from −1, to + 1, is symmetrical around zero (no effect), and the algebraic sign shows whether the effect of the manipulation is negative or positive. Because of these properties, RII enables further analysis with classical statistical methods (Armas et al., 2004)⁠. Planting of *I. glandulifera* is expected to have a negative impact on the resident vegetation, indicated by a negative RII, while removal of *I. glandulifera* is expected to have a positive effect, indicated by a positive RII. RII was applied for the above‐mentioned parameters of the resident vegetation and of *U. dioica* and the biomass of the most frequent species. For *U. dioica* individual plant biomass and stem length, RII was calculated with the mean values of 6–8 plants per plot. For each parameter, it was tested whether impact intensity RII of *I. glandulifera* planting and removal in the two habitats differs from zero using a one‐sample Wilcoxon test. Additionally, we used linear models to test whether the RII of species number, Shannon index, total biomass and vegetation height depended on the trials (planting and removal of *I. glandulifera*), the habitats (meadow and alder forest), and their interaction term.

To analyze whether plant species composition and abundance differ between the natural control situations (uninvaded or invaded) and whether *I. glandulifera* manipulations (removal or planting) have an effect on them, multivariate analyses were performed with biomass data of all species. For visualization, a nonmetric multidimensional scaling (NMDS) was performed based on Bray‐Curtis dissimilarity index (max. 80 numbers of random starts, 3 dimensions, package vegan). Differences between treatments, habitats, and their interaction were tested with PERMANOVA analyses also based on Bray‐Curtis dissimilarities (command adonis of package vegan). Study sites were given as groups within which permutations were constrained.

## 3 RESULTS

### 3.1 Dependence of *I. glandulifera* performance on treatment and habitat

**FIGURE 2:**
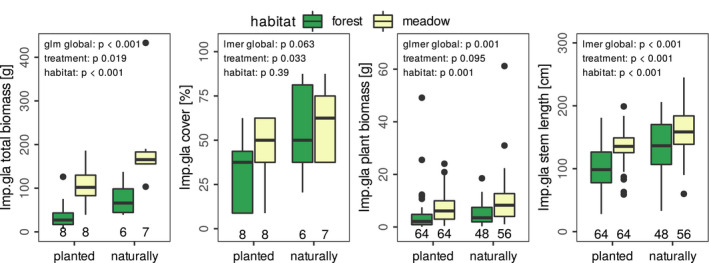
Comparison of planted and naturally grown *I. glandulifera* in the habitats alder forest and meadow. With generalized‐linear models, it was tested whether total dry biomass, cover, individual plant dry biomass, and stem length of *I. glandulifera* depended on treatment and habitat. Study site was used as random factor (lmer or glmer) unless its variance was estimated zero, thus no random factor was used (glm). For total biomass and individual plant biomass, a gamma error distribution was applied. Resulting *p*‐values are given and total sample sizes indicated at the bottom line in the graphs. Individual plant biomass and stem length *N* = 8 plants per plot

**FIGURE A1:**
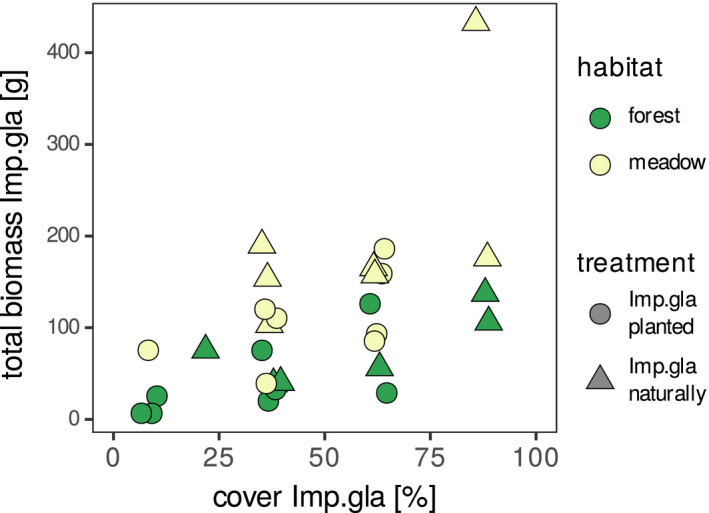
Correlation between total dry biomass and cover of *I. glandulifera*. Habitat and whether *I. glandulifera* was planted or grew naturally is indicated by point color and shape. To avoid overplotting of points, 2.5% random noise was added to *I. glandulifera* cover. Pooling both habitats and treatments, Pearson correlation coefficient was 0.726, *p* < .001. Biomass was harvested from the central 1 m^2^ per plot and given as dry weight, while cover was estimated according Braun‐Blanquet scale within the total 2.25 m^2^ plot

In the uninvaded control as in the removal treatment *I. glandulifera* remained mostly absent or occurred at very low abundances only (*I. glandulifera* dry biomass median 0.00 g, max. 0.87 g, cover less than 5%). On average 47 of the 63 planted *I. glandulifera* plants, corresponding to 74%, established. However, survival was lower in alder forests than in meadows (51% versus 85%, *p* = .012, Wilcoxon‐test). The planted *I. glandulifera* added up to a biomass of 7–186 g per plot (median 75 g, Figure 2). In natural occurrences in contrast, a higher *I. glandulifera* biomass was recorded (39–433 g, median 137 g, Figure 2). Cover of *I. glandulifera* ranged from 10% to 90% (Braun‐Blanquet classes 2a to 5) and correlated strongly with biomass (combining planted and natural occurrences, Pearson correlation coefficient r = 0.797, *p* < .001, Figure A1). Planted *I. glandulifera* plants reached similar, but slightly smaller sizes as those naturally grown (Figure 2): with 0.1–61 g biomass (median 4.8 g) plants did not differ significantly in biomass but planted ones had shorter stems than the naturally grown ones (median 126 versus 153 cm). Abundance and plant growth of both, planted and naturally grown *I. glandulifera* was lower in alder forests than in meadows (Figure 2).

### 3.2 Habitat‐dependent impact of *I. glandulifera* on the resident vegetation

**FIGURE 3:**
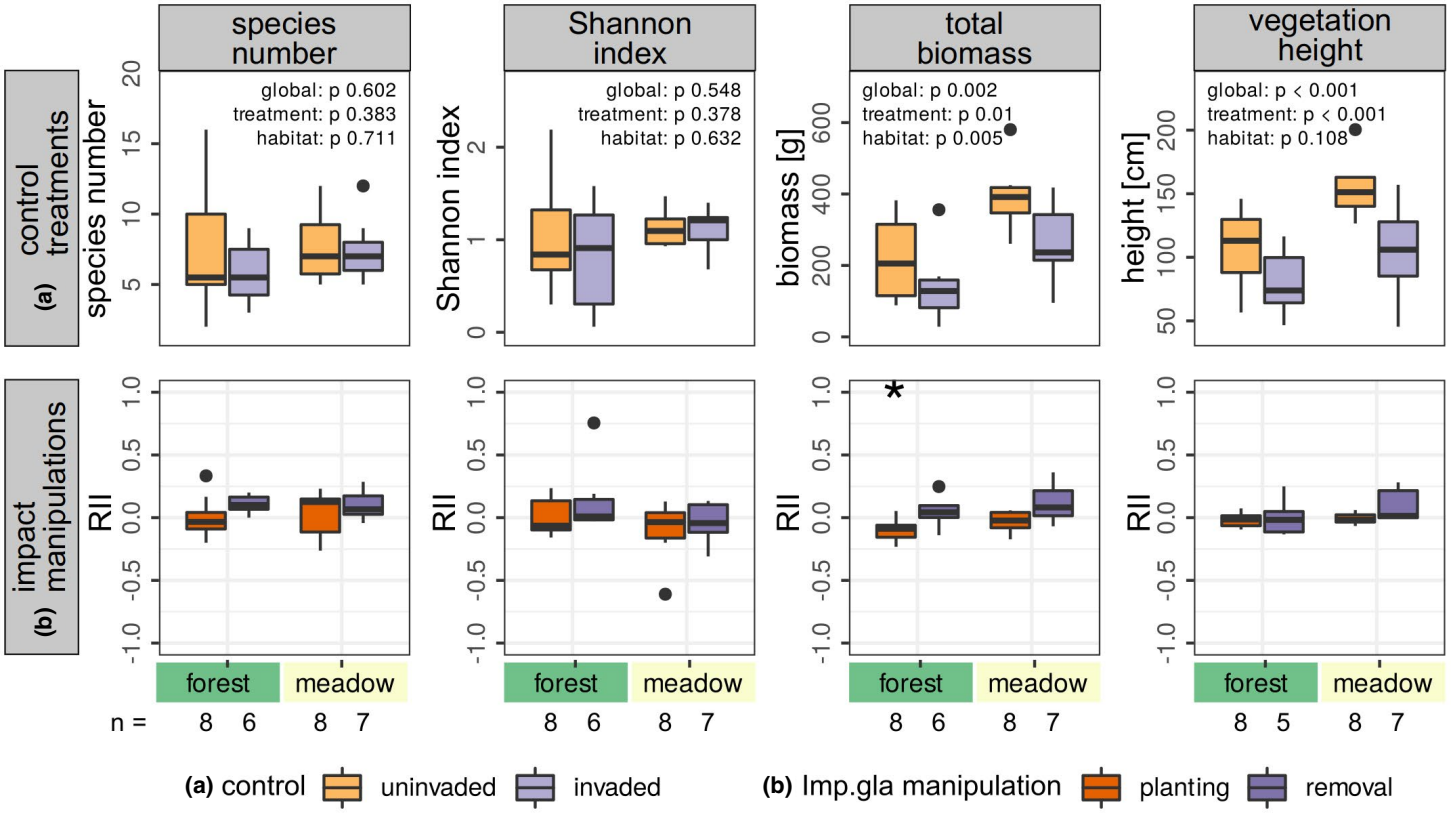
Resident vegetation characteristics in the control treatments (a) and impact intensity of *Impatiens glandulifera* planting and removal (b) depending on the habitat. With linear mixed‐effect models using site as random factor, it was tested whether the shown parameters differed between control plots invaded and uninvaded by *I. glandulifera* and between habitats (*p*‐values given). Impact intensity of *I. glandulifera* manipulation on each parameter is expressed by relative interaction index (RII) among manipulation and appropriate control per pair of plots. RII of −1 shows most negative impact, 0 no impact, and + 1 most positive impact. For planting and removal in both habitats separately, it was tested with a one‐sample Wilcoxon test whether RII differs from zero (result indicated by asterisks). Sample sizes are given at the bottom of the graphs

**FIGURE 4:**
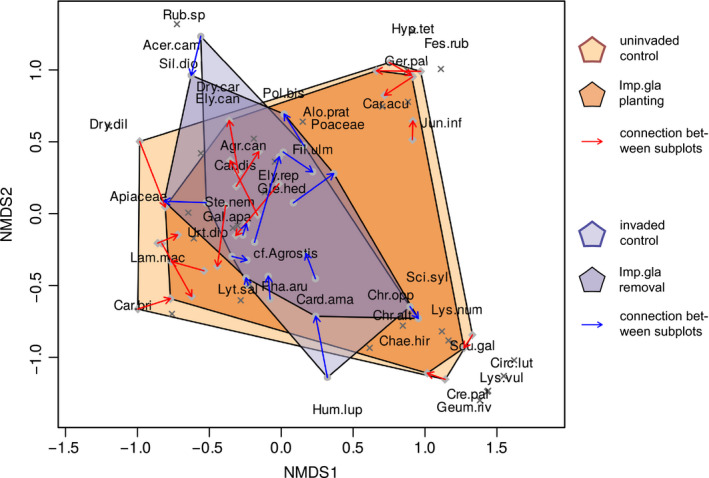
Ordination analysis of the resident species composition and abundance showing differences between invaded and uninvaded controls, respectively, and effect of *I. glandulifera* planting and removal. A nonmetric multidimensional scaling (NMDS) was performed based on Bray‐Curtis dissimilarity index of plant dry biomass, dimensions 3, stress 0.136, *n* = 58 plots. Sites are indicated by points with arrows connecting a control treatment with its corresponding plot where *I. glandulifera* was manipulated. Species are given as crosses, the most frequent ones were labeled with priority. Results of a PERMANOVA testing the differences are given in Table 1. For abbreviations of the species names see Table A1

In total 71 resident species were recorded (Table A1). Besides *I. glandulifera*, *Lamium argentatum* occurring in two pairs of plots was the only alien plant species. In the control treatments, resident species number ranged from 2 to 16 per 1 m^2^ and did not differ between invaded and uninvaded plots and between habitats, and likewise the Shannon index did not differ (Figure 3a). Total biomass and height of the resident vegetation in contrast were significantly higher in uninvaded plots than in invaded ones, biomass by about 124 g and vegetation height by almost 50 cm. Both were lower in alder forests than in meadows. However, for vegetation height, this difference was not significant because of a high variation between study sites (mixed‐effect model). Species composition and abundance differed between invaded and uninvaded plots and also between habitats (Table 1, Figure 4). For example, *Galeopsis tetrahit* and *Cardamine amara* tend to have more biomass in invaded control plots, while for *Carex acutiformis*, *Aegopodium podagraria*, and *Chaerophyllum hirsutum* this is the case in uninvaded ones. *Cirsium oleraceum*, *Ajuga reptans* and *Carex brizoides* only occurred in uninvaded control plots. Comparing habitats regarding their species composition *Geranium palustre*, *Carex acutiformis*, and *Mentha longifolia,* for example, were specific to meadows, while *Circaea lutetiana*, *Dryopteris carthusiana*, *Chrysosplenium oppositifolium*, and *Ch. alternifolium* to forests. The common species *Urtica dioica*, *Galium aparine*, *Filipendula ulmaria*, *Phalaris arundinacea*, *Stellaria nemorum*, *Agrostis caninus*, *Galeopsis tetrahit*, *Aegopodium podagraria*, and *Chaerophyllum hirsutum* occurred consistently across both habitats although biomass could vary.

**TABLE 1:** Multivariate effect of treatment and habitat on species composition and abundance, tested with a PERMANOVA based on Bray‐Curtis dissimilarities of dry biomass per species

Data subset	Coefficient	*df*	*R* ^2^	F	*p*‐value
1) Control treatments: invaded and uninvaded by *Impatiens glandulifera*	Treatment	1	0.130	4.759	.001
	Habitat	1	0.116	4.246	.001
	Treatment:habitat	1	0.070	2.565	.017
	Residual	25	0.684		
2) *Impatiens glandulifera* planting and uninvaded control	Treatment	1	0.004	0.156	.924
	Habitat	1	0.194	6.873	.831
	Treatment:habitat	1	0.012	0.421	.642
	Residual	28	0.790		
3) *Impatiens glandulifera* removal and invaded control	Treatment	1	0.019	0.516	.766
	Habitat	1	0.156	4.258	.858
	Treatment:habitat	1	0.016	0.422	.823
	Residual	22	0.809		

Note

The PERMANOVA was separately conducted for 1) the invaded and uninvaded control treatments, 2) planting trial, and 3) removal trial. Study sites were used as groups within which permutations were constrained.

A causal negative impact tested by planting or removal of *I. glandulifera* on resident vegetation characteristics was only indicated for total biomass of the resident vegetation and the impact intensity did not depend on habitat (Figure 3b, Table 2). On average, *I. glandulifera* planting decreased total resident biomass by 20 g (RII −0.08), and removal increased it by 17 g (RII + 0.08). At maximum, planting resulted in a decrease of total resident biomass from 189 to 118 g (RII −0.23) and removal of *I. glandulifera* in an increase from 95 to 203 g (RII 0.36). Pooling both habitats, RII of planting and removal on total resident biomass was different from zero (planting *p* = .016 and removal *p* = .033, one‐sample Wilcoxon test). Differing between habitats, median RII of planting on total resident biomass was negative and removal positive in both habitats, but only planting within alder forests showed a RII significantly different from zero (Figure 3b). Species composition was neither changed by *I. glandulifera* planting nor by removal, and this did not depend on habitat (Table 1, Figure 4).

**TABLE 2:** Habitat‐dependency of impact intensity (RII) of *I. glandulifera* manipulation on resident vegetation characteristics

Response	Model	Global *p*‐value	*R* ^2^	Trial	Habitat	Trial:habitat	*N*	*df*
RII species number	lm	.484	−0.017	0.207	0.67	0.735	29	3
RII Shannon index	lmer	.222		0.188	0.213	0.549	29	6
RII total biomass	lmer	**.003**		**<0.001**	0.183	0.979	29	6
RII vegetation height	lm	.106	0.124	0.66	0.84	0.272	28	3

Note

With linear models, it was tested whether the impact of *I. glandulifera* depended on trial (planting and removal of *I. glandulifera*), habitat (meadows and alder forests), and their interaction term. Study site was used as random factor (lmer) unless its variance was estimated zero, thus no random factor was used (lm). *p*‐values <.05 are given in bold.

### 3.3 Impact of *I. glandulifera* on *Urtica dioica* and other frequent species

**FIGURE 5:**
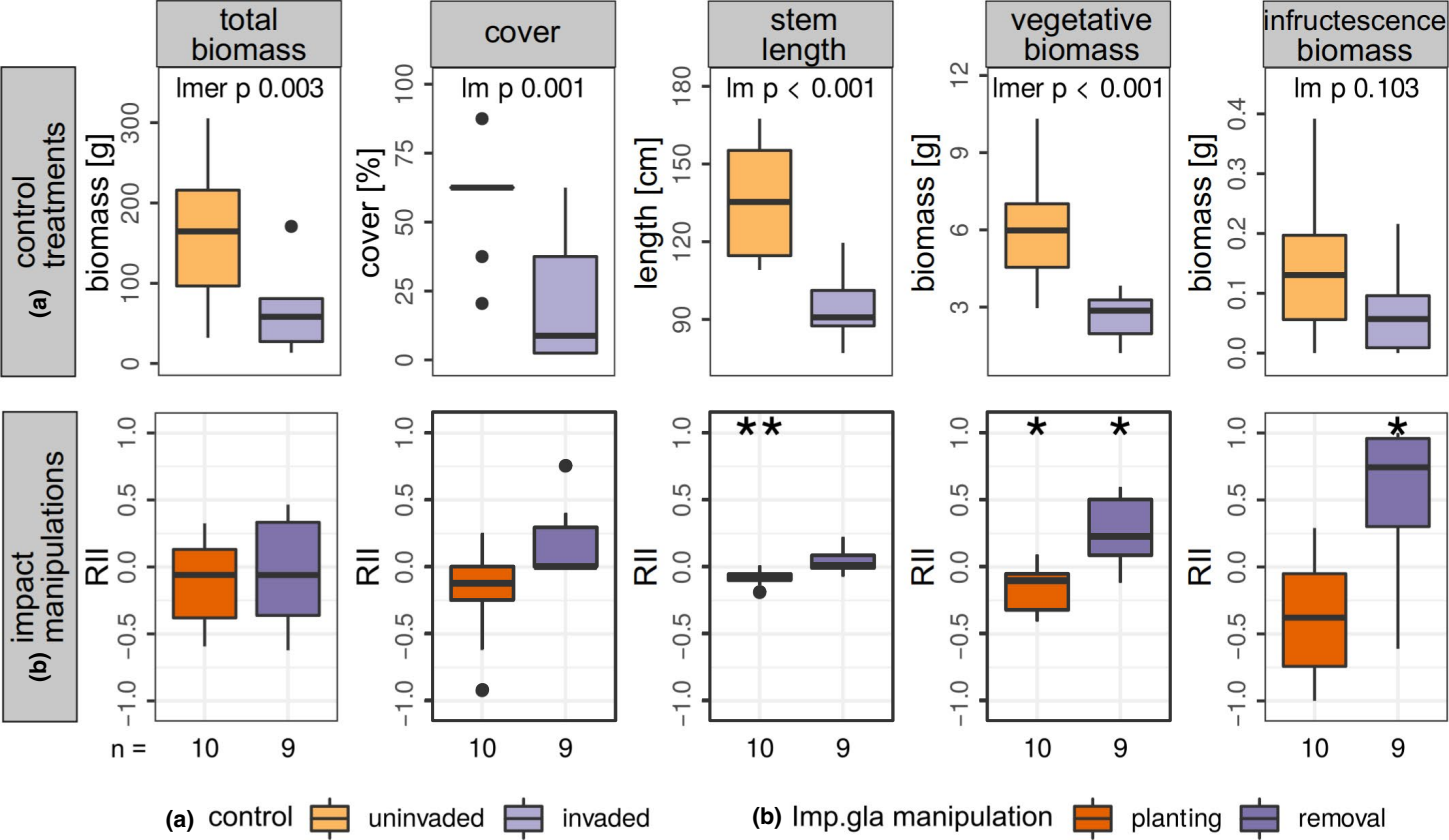
*Urtica dioica* in the control treatments (a) and impact intensity of *Impatiens glandulifera* planting and removal (b). With linear models, it was tested whether the shown parameters differed between control plots invaded and uninvaded by *I. glandulifera* (*p*‐values given). Study site was used as random factor (lmer) unless its variance was estimated zero, thus no random factor was used (lm). Impact intensity of *I. glandulifera* manipulation on each parameter is expressed by relative interaction index (RII) among manipulation and appropriate control per pair of plots. RII of −1 shows most negative impact, 0 no impact, and + 1 most positive impact. For planting and removal separately, it was tested with a one‐sample Wilcoxon test whether RII differs from zero (result indicated by asterisks). Sample sizes are given at the bottom of the graphs. Only pairs of plots are considered in which *U. dioica* occurred in both plots. Stem length, vegetative, and infructescence biomass of *U. dioica* represent mean values of 6–8 plants per plot

*Urtica dioica* grew significantly better in uninvaded than in invaded control plots regarding total biomass, cover, individual stem length, and individual vegetative biomass (Figure 5a). *U. dioica* total biomass was not changed by *I. glandulifera* manipulations while cover was slightly, but not significantly, decreased by *I. glandulifera* planting and increased by removal (Figure 5b). Individual plants of *U. dioica,* however, were affected by the manipulations regarding all considered parameters (Figure 4). Impact intensity on stem length was low but significant for planting. Impact on individual plant biomass of *U. dioica* was slightly higher. Median RII through planting was −0.11 with a maximum decrease from 6.2 to 2.6 g (RII −0.41), median RII through removal was 0.23 with a maximum increase from 1.2 to 4.8 g (RII 0.59). Impact intensity on infructescence biomass was very high but only significant in the removal trial (Figure 5b).

**FIGURE A2:**
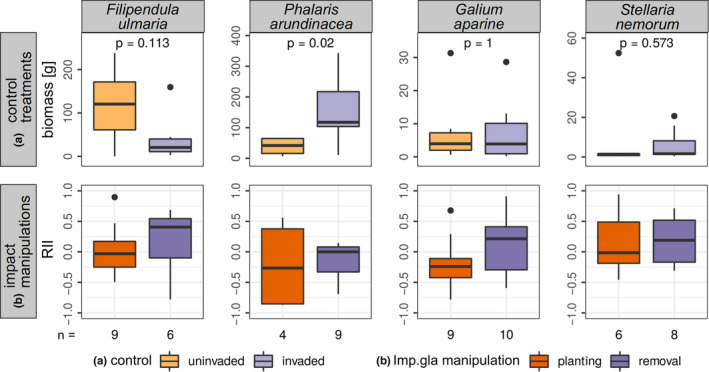
Total biomass per 1 m^2^ of the most frequent resident species in the control treatments (a) and impact intensity of *Impatiens glandulifera* planting and removal (b). With a Mann‐Whitney‐*U* test, it was tested whether the shown parameters differed between control plots invaded and uninvaded by *I. glandulifera* (*p*‐values given). Impact intensity of *I. glandulifera* manipulation on each parameter is expressed by relative interaction index (RII) among manipulation and appropriate control per pair of plots. RII of −1 shows most negative impact, 0 no impact, and + 1 most positive impact. For planting and removal separately, it was tested with a one‐sample Wilcoxon test whether RII differs from zero (result indicated by asterics). Sample sizes are given at the bottom of the graphs. Only pairs of plots are considered in which the respective species occurred in both plots

Besides *U. dioica*, the most frequent resident species were *Galium aparine*, *Filipendula ulmaria*, *Stellaria nemorum*, and *Phalaris arundinacea*. Total biomass of *P. arundinacea* was higher in invaded plots, but total biomass of the other species was independent of invaded or uninvaded situations (Figure A2a). RII of *I. glandulifera* planting and removal on each of those frequent species was highly variable and never significantly different from zero (Figure A2b). However, median total biomass of *G. aparine* decreased by planting and median total biomass of *F. ulmaria*, *G. aparine*, and *S. nemorum* increased by removal.

## 4 DISCUSSION

In this field study, we experimentally removed *Impatiens glandulifera* from invaded plots, and planted *I. glandulifera* in formerly uninvaded plots in order to test whether *I. glandulifera* has a negative impact on the native vegetation in riparian meadows and alder forests, and whether the impact is causal or not. We found that *I. glandulifera* had a causal impact indicated by a negative effect of planting and a positive effect of removal of *I. glandulifera* on total resident biomass and individual plant growth of *Urtica dioica* but not on α‐diversity, species composition, vegetation height, and total biomass of the most frequent co‐occurring species. Impact of the manipulations depended only slightly on the habitat.

### 4.1 *Impatiens glandulifera* had low but causal impact on native vegetation

Removal of *Impatiens glandulifera* had a positive and planting a negative effect on total resident plant biomass and growth of *Urtica dioica* individual plants. This indicates that *I. glandulifera* is a driver of ecosystem changes having a causal negative impact on the resident vegetation. A causal impact of *I. glandulifera* on native vegetation is also indicated by Hejda and Pyšek (2006), Hulme and Bremner (2006), and Cockel et al., (2014) who all found positive, but often only slight effects of *I. glandulifera* removal on riparian plant species diversity and composition, which were, however, not affected in the present study. A causal impact on *U. dioica* plants as found in the present study is underpinned by experimental studies on competitive and allelopathic interactions of both species (Bieberich et al., 2018; Gruntman et al., 2014; Tickner et al., 2001)⁠. However, the impact of *I. glandulifera* on *U. dioica* competing in a pot experiment was much stronger (relative interaction index RII about −0.7, in Gruntman et al., (2014)⁠ and Bieberich et al., (2018)) than under the field conditions in the present study (median RII planting −0.09). Taken together the impact of *I. glandulifera* can be rated as low. Total resident biomass and individual plant growth of *U. dioica* were affected by planting and removal indeed, but only to a small extend, and α‐diversity, species composition, vegetation height, and total biomass of the most frequent co‐occurring species were not affected by the manipulations at all.

Criteria of a clear driver of changes were only partially met for *I. glandulifera* in the present study. If the species was a clear driver, planted *I. glandulifera* should establish and clearly suppress natives, while removal would lead to recovery of the native vegetation (Bauer, 2012; Didham et al., 2005; MacDougall & Turkington, 2005)⁠. In the present study, planted *I. glandulifera* reached similar, but slightly smaller sizes than naturally growing ones. Establishment and growth of *I. glandulifera* were lower in alder forests than in meadows. Thus, *I. glandulifer*a growth was slightly restricted by resident vegetation and native plant species α‐diversity was not affected at all. Species composition, vegetation height, *U. dioica* total biomass, and *U. dioica* cover differed among invaded and uninvaded plots. However, they were not subsequently affected by removal and planting of *I. glandulifera*. On the one hand, this can indicate that differences between invaded and uninvaded plots were not caused by *I. glandulifera* but are due to other factors, such as habitat conditions or disturbances. If these factors already differed between plots before *I. glandulifera* invasion, they themselves could be one reason for the invasion success at a particular patch. In this case, only comparing invaded and uninvaded patches observationally could lead to the false conclusion that *I. glandulifera* has a negative impact on native vegetation. On the other hand, a response of the native vegetation to the *I. glandulifera* manipulations indicating a causal effect could take longer time than the study duration of one season (Cockel et al., 2014; Rusterholz et al., 2017). Also between‐year variations could obscure long‐term effects. However, the manipulations affected total native biomass and performance of *U. dioica*, the response of which is faster and more sensitive in comparison with diversity measures. This indicates a fast competitive and allelopathic effect on the growth of neighboring plants as known for the annual *I. glandulifera* from the seedling stage onwards (Bieberich et al., 2020; Gruntman et al., 2014). Another limitation of this experimental study design is that the removal and planting of any other plant species could have the same effect as the removal and planting of *I. glandulifera*, and thus the results might not be specific to *I. glandulifera*. However, results of the present study are corroborated by a previous observational study within the same sites, which underpins that *I. glandulifera* has no impact on α‐diversity, species composition, and vegetation height, but on abundance of *U. dioica* (Bieberich et al., 2020). We suggest that continuing the manipulations for more than one season may lead to a change of total abundance of *U. dioica* as a consequence of the reduced growth of individual plants.

If *I. glandulifera* is not a strict driver of changes, it could be a back‐seat driver, whose invasion is favored by previous ecosystem changes until it becomes a driver of further changes itself (Bauer, 2012). Affinity of *I. glandulifera* to habitats with natural and anthropogenic disturbances and changed land use (Ammer et al., 2011; Beerling & Perrins, 1993; Čuda, Rumlerová, et al., 2017; Čuda et al., 2020; Pyšek & Prach, 1993, 1995) also indicates characteristics of a back‐seat driver. However, to clearly distinguish a back‐seat driver from a driver is not possible with the present study. To this end, it would be necessary to test whether removal of the invader would result in recovery of the initial state of an ecosystem only in combination with habitat restoration (Bauer, 2012).

### 4.2 Causal impact of *I. glandulifera* depended only slightly on the habitat

We found a consistent effect of *I. glandulifera* manipulations on native vegetation in alder forests and meadows: In both habitats, *I. glandulifera* caused a reduction of total resident biomass but had no causal impact on species composition, α‐diversity, and vegetation height. According to a linear model, RII on total biomass did not differ between the two habitats, alder forests and meadows. However, there was a small difference between habitats, as the RII on total biomass was significantly different from zero in alder forests but not in meadows in the *I. glandulifera* planting trial. This indicates a higher impact in elder forests, where both, the biomass of *I. glandulifera* and the resident vegetation was lower than in meadows. In contrast, in our previous study within the same study sites, we found negative correlations between cover of *I. glandulifera* and cover of *U. dioica*, *F. ulmaria* and total cover, which were stronger under bright conditions with higher *I. glandulifera* cover than under dark site conditions (Bieberich et al., 2020). Comparing invaded and uninvaded sites, also Diekmann et al., (2016) suggested a higher impact of *I. glandulifera* in open than in more shady habitats. Thus, the correlative impact seems to be stronger habitat‐dependent than the short‐time causal impact and more pronounced in bright habitats.

### 4.3 Implications for assessment of impact and for nature conservation

We found that the impact of *I. glandulifera* on native vegetation was causal but low. The response of the native vegetation to the *I. glandulifera* manipulations was quite fast within one vegetation period, even if only some parameters were affected within the study duration. Also other field studies on *I. glandulifera* using a removal approach found effects on native vegetation within one season (Cockel et al., 2014; Hejda & Pyšek, 2006; Hulme & Bremner, 2006),⁠ whereas only in Hulme and Bremner (2006), the effect was quite high. This means that invasion can have a negative impact after a short period of time, but also removal as management measure could have a fast effect. However, the impact of *I. glandulifera* could also increase over time after invasion (Rusterholz et al., 2017), and longer lasting removal can also enhance a management effect (Cockel et al., 2014; Rusterholz et al., 2017).

We suggest that *I. glandulifera* is not a clear driver of changes, but it has some characteristics of a back‐seat driver benefiting from previous changes such as disturbances or changed land use. This is relevant for nature conservation because drivers and back‐seat drivers require a different management strategy. In the case of a driver, removal of the invader, which induced the changes, is ideally sufficient (Bauer, 2012). In contrast, in the case of a back‐seat driver, habitat restoration is necessary in addition to removal of the invader (Bauer, 2012). Thus, management of a back‐seat driver is more complicated because the previous changes that facilitated invasion have to be known and countered. Such previous changes can be all kinds of alterations of ecosystem properties such as land use change, pollution, nutrient input, or altered disturbance regimes (Bauer, 2012; Didham et al., 2005). Unfortunately, there is often no reliable information on the original community and ecosystem processes available (Parker et al., 1999). Special cases are natural disturbances and intentional anthropogenic ecosystem changes. Natural and anthropogenic disturbances are common in riparian habitats and can generally favor invasions (Richardson et al., 2007). Intentional ecosystem changes such as tree cutting or habitat restoration are sometimes associated with *I. glandulifera* invasion (for forests: Čuda et al. (2020), river restoration: Lapin et al. (2016)). In this case, it can be recommended to prevent the potential invasion of a back‐seat driver while planning and conducting the disturbance (D’Antonio & Meyerson, 2002; Lapin et al., 2016)⁠. It is also possible that *I. glandulifera* invasions are favored by anthropogenic nutrient input as *I. glandulifera* has an affinity to nutrient‐rich patches (Bieberich et al., 2020; Čuda et al., 2014)⁠. Thus, reducing the nutrient input into water bodies as a general aim of nature conservation may also reduce invasion of *I. glandulifera*. In the case of already established populations of *I. glandulifera,* it can be discussed if a management is reasonable, considering the rather low impact of *I. glandulifera* in combination with its high abundance and frequency in Central Europe. Since a population control can be very expensive (Leblanc & Lavoie, 2017), it should be reserved for sites which are particularly valuable in terms of nature conservation.

## 5 CONCLUSION

*Impatiens glandulifera* had a causal but low impact on the resident vegetation in both riparian habitats, alder forests and meadows. The effect could be seen already after one season, but may also intensify over longer time. *Impatiens glandulifera* had some characteristics of a back‐seat driver, which is facilitated by previous ecosystem changes but is also a driver of further changes having causal impact on the invaded ecosystem. If *I. glandulifera* has to be managed for nature conservation, this involves the need of ecosystem restoration along with removal of the invader.

## CONFLICT OF INTEREST

The authors declare that there are no conflicts of interest.

## AUTHOR CONTRIBUTION

**Judith Bieberich:** Conceptualization (equal); Data curation (lead); Formal analysis (lead); Investigation (lead); Methodology (equal); Project administration (equal); Visualization (lead); Writing‐original draft (lead); Writing‐review & editing (equal). **Stefanie Müller:** Formal analysis (supporting); Investigation (supporting); Methodology (equal); Visualization (supporting); Writing‐original draft (supporting); Writing‐review & editing (supporting). **Heike Feldhaar:** Conceptualization (equal); Methodology (equal); Project administration (equal); Resources (supporting); Supervision (equal); Writing‐review & editing (equal). **Marianne Lauerer:** Conceptualization (equal); Methodology (equal); Project administration (equal); Resources (lead); Supervision (equal); Writing‐review & editing (equal).

## Data Availability

All data of the manuscript are available at Dryad (https://doi.org/10.5061/dryad.59zw3r25z).

## Appendix

**TABLE A1:** Abbreviations of species names as shown in Figure 4 and number of plot in which each species occurred

Abbreviation	Taxon name according to the determinable level	Occurrences
Acer.cam	*Acer campestre L*.	1
Acer.pse	*Acer pseudoplatanus L*.	5
Aeg.pod	*Aegopodium podagraria L*.	11
Agr.can	*Agrostis canina L*. (*incl. A. cf. canina L*.)	12
Aju.rep	*Ajuga reptans L*.	4
All.pet	*Alliaria petiolata* (*M. Bieb*.) *Cavara et Grande*	6
Alo.prat	*Alopecurus pratensis L*.	11
Ang.syl	*Angelica sylvestris L*.	1
Apiaceae	*Apiaceae*	1
Ath.fil	*Athyrium filix‐femina (L.) Roth*	3
Cal.sep	*Calystegia sepium (L.) R. Br*.	2
Calt.pal	*Caltha palustris L*.	4
Car.acu	*Carex acutiformis Ehrh*.	15
Car.bri	*Carex brizoides L*.	4
Car.dis	*Carex disticha Huds*.	2
Card.ama	*Cardamine amara L*.	7
cf.Agrostis	probably *Agrostis*	2
Chae.hir	*Chaerophyllum hirsutum L*.	10
Chr.alt	*Chrysosplenium alternifolium L*.	3
Chr.opp	*Chrysosplenium oppositifolium L*.	5
Cir.ole	*Cirsium oleraceum (L.) Scop*.	6
Circ.int	*Circaea intermedia Ehrh*.	1
Circ.lut	*Circaea lutetiana L*.	2
Cre.pal	*Crepis paludosa L. Moench*	4
Dac.glo	*Dactylis glomerata L*.	2
Des.ces	*Deschampsia cespitosa (L.) P. Beauv*.	2
Dry.car	*Dryopteris carthusiana (Vill.) H.P. Fuchs*	4
Dry.dil	*Dryopteris cf. dilatata (Hoffm.) A. Gray*	1
Ely.can	*Elymus caninus (L.) L*.	1
Ely.rep	*Elymus repens (L.) Gould*	1
Epi.ang	*Epilobium angustifolium L*.	1
Epi.sp	*Epilolium sp*.	1
Equi.flu	*Equisetum fluviatile L*.	8
Fes.rub	*Festuca rubra L*.	2
Fil.ulm	*Filipendula ulmaria (L.) Maxim*.	36
Fra.exc	*Fraxinus excelsior L*.	4
Gal.apa	*Galium aparine L*.	42
Gal.elo	*Galium elongatum C. Presl*	1
Gal.mol	*Galium molugo L*.	1
Gal.tet	*Galeopsis tetrahit L*.	11
Ger.pal	*Geranium palustre L*.	8
Geum.riv	*Geum rivale L*.	2
Geum.urb	*Geum urbanum L*.	7
Gle.hed	*Glechoma hederacea L*.	7
Hum.lup	*Humulus lupulus L*.	1
Hyp.tet	*Hypericum tetrapterum Fr*.	1
Imp.nol	*Impatiens noli‐tangere L*.	2
Jun.inf	*Juncus inflexus L*.	1
Lam.arg	*Lamium galeobdolon ssp. argentatum (Smejkal) Duvigneau*	3
Lam.mac	*Lamium maculatum (L.) L*.	6
Lat.pra	*Lathyrus pratensis L*.	2
Lys.num	*Lysimachia nummularia L*.	5
Lys.vul	*Lysimachia vulgaris L*.	3
Lyt.sal	*Lythrum salicaria L*.	1
Men.lon	*Mentha longifolia (L.) L*.	13
Myo.sco	*Myosotis scorpioides L*.	2
Pha.aru	*Phalaris arundinacea L*.	30
Poa.ang	*Poa angustifolia L*.	1
Poaceae	*Poaceae*	7
Pol.bis	*Polygonum bistorta L*.	7
Pri.ela	*Primula cf. elatior (L.) Hill*	1
Pru.pad	*Prunus padus L*.	1
Rub.sp	*Rubus sp*.	3
Rum.obt	*Rumex obtusifolius L*.	1
Sci.syl	*Scirpus sylvaticus L*.	3
Scu.gal	*Scutellaria galericulata L*.	2
Sil.dio	*Silene dioica (L.) Clairv*.	2
Ste.nem	*Stellaria nemorum L*.	30
Urt.dio	*Urtica dioica L*.	41
Val.dio	*Valeriana dioica L*.	3
Vib.opu	*Viburnum opulus L*.	1
